# Nobody’s perfect: can irregularities in pit structure influence vulnerability to cavitation?

**DOI:** 10.3389/fpls.2013.00453

**Published:** 2013-11-12

**Authors:** Lenka Plavcová, Steven Jansen, Matthias Klepsch, Uwe G. Hacke

**Affiliations:** ^1^Institute for Systematic Botany and Ecology, Ulm UniversityUlm, Germany; ^2^Department of Renewable Resources, University of AlbertaEdmonton, AB, Canada

**Keywords:** bordered pit, pit damage, pit membrane, pit ontogeny, structural irregularity, xylem vulnerability

## Abstract

Recent studies have suggested that species-specific pit properties such as pit membrane thickness, pit membrane porosity, torus-to-aperture diameter ratio and pit chamber depth influence xylem vulnerability to cavitation. Despite the indisputable importance of using mean pit characteristics, considerable variability in pit structure within a single species or even within a single pit field should be acknowledged. According to the rare pit hypothesis, a single pit that is more air-permeable than many neighboring pits is sufficient to allow air-seeding. Therefore, any irregularities or morphological abnormalities in pit structure allowing air-seeding should be associated with increased vulnerability to cavitation. Considering the currently proposed models of air-seeding, pit features such as rare, large pores in the pit membrane, torus extensions, and plasmodesmatal pores in a torus can represent potential glitches. These aberrations in pit structure could either result from inherent developmental flaws, or from damage caused to the pit membrane by chemical and physical agents. This suggests the existence of interesting feedbacks between abiotic and biotic stresses in xylem physiology.****

## INTRODUCTION

Adjacent xylem conduits in both gymnosperms and angiosperms are joined by common endwalls. In order to facilitate connectivity, the endwalls are permeable to water and dissolved substances, making the xylem network well suited for the long-distance transport of water, nutrients, and signaling molecules. A certain degree of xylem network compartmentalization is, however, desirable because it helps to confine the spread of air embolism and xylem-borne pathogens. The conflicting needs for the connectivity and isolation within the xylem network have been ingeniously solved by the evolution of endwall pitting.

Bordered pits in water-conducting xylem cells have a characteristic structure of two basic components – the pit membrane and the pit border. Pit membranes are uniformly thick and porous in most angiosperms, whereas two distinct regions – a thicker solid torus and a thinner highly porous margo – are characteristic for the pit membranes of gymnosperms. While pits shares common features in their overall structure, the finer-scale characteristics such as the pit size, pit membrane thickness, pit chamber depth, torus to aperture overlap vary substantially between different species (Jansen et al., 2009; Pittermann et al., 2010) as well as within the same species depending on growing conditions (Schoonmaker et al., 2010; Plavcová et al., 2011) or the position within a plant (Domec et al., 2008; Hacke and Jansen, 2009).

**FIGURE 1:**
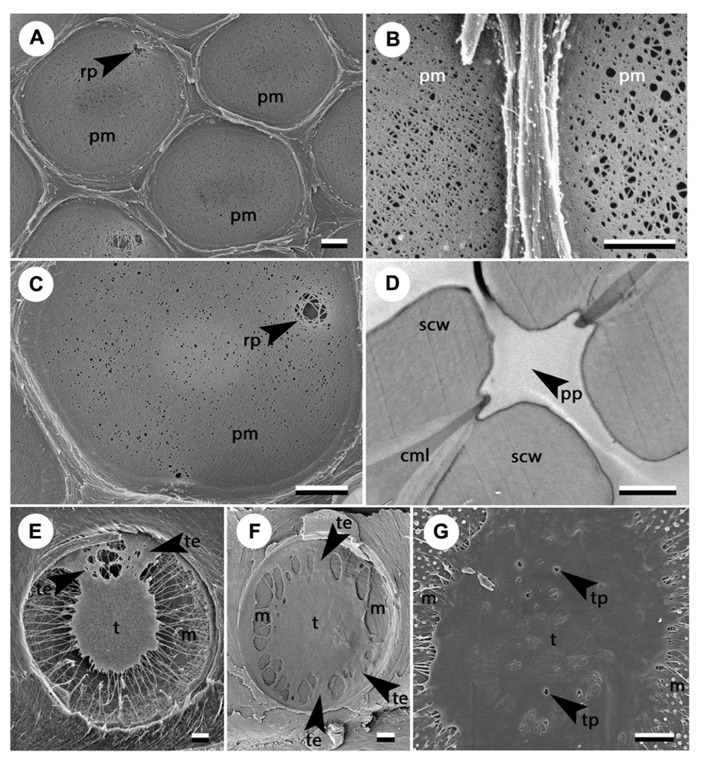
**Structural variability in adjacent pits (A, B) and examples of profound irregularities in pit structure (C–E).** **(A)** Pit field in *Acer negundo* in which some pit membranes are homogenously porous while others show rare large pores (photo courtesy of Brendan Choat); **(B)** adjacent pits in hybrid poplar *Populus trichocarpa* × *deltoides* showing different average porosity; **(C)** rare large pore independent of the average porosity in *Acer negundo* (photo courtesy of B. Choat); **(D)** perforated pit in a non-conductive ITE in *Lycium andersonii*; **(E)** sporadic torus extensions in *Picea mariana *(photo courtesy of Amanda Schoonmaker); **(F)** numerous torus extensions in *Widdringtonia cederbergensis*; **(G)** punctured torus in *Sequoia sempervirens*. Scale bars show 1 μm. Arrowheads indicate irregularities in the pit structure. cml, compound middle lamella; m, margo; pm, pit membrane; pp, perforated pit; rp, rare large pore; scw, secondary cell wall; t, torus; te, torus extension; tp, torus perforation.

Looking for links between pit structure and embolism resistance has become very topical in the field of xylem hydraulics. Most studies so far have focused on the structure of a typical pit and assessed parameters such as mean pit membrane thickness, mean pit membrane porosity, or mean torus to aperture overlap. However, considerable variation in the structure of pits exists even within the same pit field (**Figures 1A,B**). Moreover, some pits exhibit remarkable structural aberrations, including particularly large pores independent of mean porosity in angiosperms (**Figures 1A,C**) and punctured or irregular tori in gymnosperms (**Figures 1E–G**). Arguably these pits, which represent the tails of, or even outliers in, the overall pit distribution, matter the most for the spread of embolism (Christman et al., 2009).Thus, the aim of this paper is (1) to highlight the existence of irregularities in pit structure that can have substantial influence on their permeability to air, and (2) to review possible mechanisms that can give rise to such irregularities.

## DEVELOPMENTAL IRREGULARITIES OF PIT MEMBRANES

The ontogeny of pits is a complex process consisting of several steps. Pit development is initiated by the delineation of their outlines in the primary cell wall (Wardrop, 1954; Imamura and Harada, 1973), followed by the gradual deposition of secondary wall constituting pit borders (Chaffey et al., 1997) and the extensive hydrolysis and remodeling of pit membranes (Czaninski, 1972; Butterfield and Meylan, 1982; Kim et al., 2011).

In angiosperms, the selective hydrolysis of pit membranes is a critical step affecting their final porosity. While the chemical composition of pit membranes remains poorly characterized (Choat et al., 2008), it is generally assumed that most of the non-cellulosic components are removed before xylem conduits reach maturity (O’Brien and Thimann, 1967; Czaninski, 1972). Depending on the thickness of the pit membrane and the amount of amorphous material left after the hydrolysis, some pit membranes show pores embedded within the fibrillar meshwork, whereas other membranes appear non-porous when observed with scanning electron microscope (SEM; Jansen et al., 2009). In multi-cellular vessels, the primary cell wall of some endwalls is hydrolyzed completely, giving rise to perforation plates (Benayoun et al., 1981). Interestingly, transitional cases between scalariform perforation plates and pitted endwalls are sometimes found in ferns (Carlquist and Schneider, 1997, 2001) and basal groups of vessel-bearing angiosperms (Carlquist, 1992; Sperry et al., 2007). The pit membranes of vesselless angiosperms typically exhibit high porosity (Hacke et al., 2007). Furthermore, pits between imperforate tracheary elements (ITEs) provide an interesting comparison to intervessel pit membranes. In a recent study, Sano et al. (2011) showed that there is a clear difference in the integrity of the pit membrane depending on the ITE’s function. While homogenously porous pit membranes are present in conductive ITEs, pit membranes are often perforated in non-conductive ITEs (**Figure 1D**). However, it is not clear at what stage of their development the membranes of non-conductive ITEs become perforated, or what mechanisms are responsible. Taken together, these observations suggest that the hydrolytic machinery involved in the development of intervessel pit membranes must have been painstakingly fine-tuned over the course of evolution in order to produce the relatively efficient and safe vessel-based xylem of extant eudicots.

For the same reason, it seems unlikely that such an extensive and complex hydrolytic process is always flawless. One can easily imagine that a local excess of hydrolytic activity or a slight irregularity in the thickness or arrangement of the primary cell wall could give rise to a particularly large pore within the pit membrane (**Figures 1A,C**). This idea is consistent with the “rare pit” hypothesis (Hargrave et al., 1994; Choat et al., 2003; Wheeler et al., 2005; Christman et al., 2009). According to this hypothesis, a small portion of pits contain a large pore, while the vast majority of pits have much narrower, air-tight pores. Exceptionally large pores are occasionally observed with SEM (**Figures 1A,C**; Sano, 2005) independent of the average membrane porosity; however, it remains uncertain whether these pores are real, or artifacts resulting from sample preparation. The existence of rare leaky pits has been nevertheless supported by particle perfusion experiments (Choat et al., 2003), air-seeding experiments (Christman et al., 2009, 2012), and observed correlations between intervessel pit area and vulnerability to cavitation (Wheeler et al., 2005; Hacke et al., 2006).

The deposition of secondary material resulting in the development of a torus represents a characteristic step in the ontogeny of pits in gymnosperms. The secondary layer is initially deposited over the entire surface of the pit membrane, with its thickness being greatest in the center of the membrane. Subsequently, some of the material is removed by autolysis, which exposes the highly porous margo and defines the final shape and thickness of the torus (Dute, 1994; Dute et al., 2008). Tori are usually depicted as round symmetrical discs. However, tori of many pits show structural irregularities such as scalloped tori and torus extensions (**Figures 1E,F**). Tori with scalloped margins are characteristic of *Cedrus *but can occasionally be found in other Pinaceae and Cupressaceae (Richter et al., 2004). Torus extensions are relatively common among gymnosperms, although their occurrence is not frequently highlighted. It is imaginable that these irregularities can potentially prevent tori from properly sealing the apertures. In support of this hypothesis, Schoonmaker et al. (2010) found a higher occurrence of torus extensions in more vulnerable xylem of *Picea mariana* grown in an understory in comparison with less vulnerable trees grown in an open field. In contrast, Pittermann et al. (2010) found that torus extensions were more frequent in cavitation resistant species of Cupressaceae.

Punctured tori (**Figure 1G**) represent another irregularity in the structure of gymnosperm pits that appears to be associated with increased vulnerability (Jansen et al., 2012). Punctured tori frequently occur in Pinaceae but are also observed in some members of Cupressaceae and Cephalotaxaceae (Jansen et al., 2012). These “imperfections” likely arise from the combination of plasmodemata present during early developmental stages of the torus, and the lack of matrix removal from the torus (as observed for instance in *Abies firma*, but not in *Metasequoia glyptostroboides*, Dute et al., 2008).

In summary, the ontogeny of pits in both angiosperms and gymnosperms is a complex process and the mechanisms regulating this process are not well understood. Obtaining more detailed knowledge of the hydrolytic machinery involved in the remodeling of pit membranes would be particularly useful as rare large pores, irregularities of torus margins, and punctured tori could be underpinned by variation in the hydrolytic activity exerted during the pit membrane development.

## DAMAGE TO PIT MEMBRANES BY PHYSICAL AND CHEMICAL AGENTS

With a typical thickness between 100 and 300 nm, pit membranes represent delicate structures likely prone to structural damage by physical or chemical stress, or a combination of the two. Extensive disruption of pit membranes has been documented with SEM in several studies. Harvey and van den Driessche (1997) observed a higher occurrence of ripped and torn membranes in drought susceptible poplar clones. Sperry et al. (1991) showed that pit membrane degradation and increased vulnerability to cavitation are concomitants of wood senescence in trembling aspen. More recently, pit deterioration has also been documented in grapevine stems infested with the bacterial pathogen *Xylella fastidiosa* (Sun et al., 2011). While these studies portray extreme situations, they illustrate what may commonly occur *in planta*, although to a more moderate extent. Choat et al. (2003) highlighted some of these studies and suggested that occasional damage to pit membranes could represent an important source of air-seeding sites. Here we elaborate upon this hypothesis and review possible mechanism potentially causing pit membrane damage.

Under field conditions, substantial mechanical forces can be generated by factors such as wind, snow-load, hail, or rough handling by animals; e.g., large mammalian herbivores bending a twig while chewing on leaves (Cannell and Morgan, 1989; Lucas et al., 2000; Rudnicki et al., 2001). Wood typically exhibits sufficient mechanical strength not to break under these types of loading. However, it is possible that the perturbations are sufficient to deform the pit fields and cause occasional damage to the pit membranes, especially in younger branches. Close correlations between xylem vulnerability and wood density or conduit wall thickness are frequently observed (e.g., Hacke et al., 2001a; Fichot et al., 2010; Plavcová and Hacke, 2012; Lens et al., 2013). It is possible that stronger mechanical reinforcement helps to prevent pit membrane damage caused by external mechanical stress.

Alternatively, this reinforcement may be required to avoid conduit collapse caused by negative xylem pressure (Hacke et al., 2001a; Jacobsen et al., 2005). The implosion of xylem conduits has not been frequently observed except in xylem with a severely perturbed deposition of cell wall lignin (Barnett, 1977; Turner and Somerville, 1997; ), suggesting that air-seeding typically occurs before the xylem pressure reaches the implosion limit. This raises yet another important question, namely, what is the effect of the pressure difference exerted on the pit membranes during and shortly before air-seeding. When subjected to internal mechanical stress, pit membranes deflect, and stretch. This may cause transient or permanent changes in pit membrane porosity (Choat et al., 2004) and eventually lead to membrane rupture (Sperry and Hacke, 2004; Domec et al., 2006). There is also evidence that xylem becomes more vulnerable as a result of a previous cavitation event. This “cavitation fatigue” phenomenon has been observed in two poplar species (*Populus* *tremuloides* and *P. angustifolia*), sunflower (*Helianthus annuus*), and chestnut petioles (*Aesculus hippocastanum*; Hacke et al., 2001b; Stiller and Sperry, 2002; Anderegg et al., 2013). It was hypothesized that increased air permeability in weakened xylem results from rupture or loosening of pit membrane fibrils (Hacke et al., 2001b), but direct evidence for this idea is still lacking.

In spite of the general perception that pit membranes are fragile, no permanent differences in the permeability of pit membranes to colloidal gold were observed after applying injection pressures of up to 6 MPa (Choat et al., 2004). Furthermore, Michaletz et al. (2012) demonstrated that pit membranes of *Populus balsamifera* are durable under conditions of extreme heat. Exposure to a temperature of 65°C did not change pit membrane permeability to air, despite causing severe deformations of conduit walls. However, this result originates from a laboratory experiment and it is possible that additional perturbations can cause pit membrane damage more easily in xylem weakened by the heat. Such situation may readily occur in the field; e.g., as a forest stand is exposed to more wind following forest fire.

Furthermore, the integrity of pit membranes can be disrupted by chemical agents reacting with the structural components of the membrane. Besides water, xylem sap contains a wide array of chemicals, some of which are known to induce the loosening of plant cell walls. Among these are numerous polysaccharide-hydrolyzing enzymes. The destructive effect of cellulase on pit membranes has been demonstrated by a marked increase in xylem hydraulic conductivity following the hydrolytic treatment (Schulte et al., 1987). Under natural conditions, pit membranes weakened by a hydrolytic reaction may serve as gateway for pathogens colonizing the xylem. While some viruses such as the tobacco rattle virus can spread through pit membranes without the need to widen the pores (Garbaczewska et al., 2012), larger bacteria must perturb the structure of the pit membranes in order to penetrate the adjacent conduit (Pérez-Donoso et al., 2010). For instance, *Xyllela fastidiousa*, which causes the Pierce’s disease in grapevine, likely**disrupts pit membranes by secreting a cocktail of hydrolytic enzymes including β-1,4-endoglucanses, xylanases, xylosidases, and polygalacturonases (Roper et al., 2007; Pérez-Donoso et al., 2010). Another bacterium, *Pseudomonas fluorescens*, has been shown to degrade torus-margo pits in pine wood chips. While the margo region underwent substantial degradation, the torus region remained largely intact, demonstrating the differential resistance of these pit membrane regions to microbial degradation (Burnes et al., 2000). Fungal infestations are also known to cause pit membrane degradation, with most observations being done on dead decaying wood (Schwarze et al., 2006). There is a plethora of fungal pathogens infecting the sapwood of standing trees (e.g., blue stain fungi, root- and trunk-rots); however, little is known about their actual effects on xylem hydraulics.

Another way to alter the pore size in pit membranes is the depletion of Ca^2^^+^. Calcium-pectin complexes are presumably present throughout the entire surface of the pit membrane (Zwieniecki et al., 2001; Gortan et al., 2011) or at least in a restricted part of the membrane (Plavcová and Hacke, 2011). Indirect evidence that calcium removal causes enlargement of pit membrane pores has been provided by Sperry and Tyree (1988) and by Herbette and Cochard (2010) who showed that the perfusion of xylem with calcium-chelating agents such as oxalic acid, EGTA, and phosphate buffer results in increased vulnerability to cavitation. In a similar experiment, the stems of *Drymis winteri *were treated with a calcium-chelating phosphate buffer, which resulted in an increase in the mean pit membrane porosity from 6 to 120 nm as measured with SEM (Klepsch et al., unpublished). Furthermore, it has been suggested that the rice yellow mottle virus extracts Ca^2^^+^ from the pit membrane and incorporates it in its own structures. The result is a loosening of pectin hydrogels and widening of pit membrane pores, in turn allowing systemic virus transport via xylem conduits (Opalka et al., 1998).

Reactive oxygen species (ROS) represent another type of chemical agent potentially capable of disrupting pit membranes, although this hypothesis has not yet been tested. ROS are commonly found in xylem sap and their concentration increases as a result of biotic or abiotic stress (Wang et al., 2008). The oxidative activity of ROS is known to induce cleavage of cell wall polysaccharides *in vivo* during normal plant growth and development (Schopfer, 2001; Müller et al., 2009). Moreover, high concentrations of ROS may lead to severe oxidative damage. For instance, in two pea (*Pisum sativum*) cultivars subjected to saline stress, necrotic leaf lesions were induced in close proximity of minor veins and could be linked with the elevated ROS concentration in the leaf apoplast (Hernández et al., 2001).

Thus, more research is still needed to demonstrate whether mature pit membranes stay intact throughout the entire functional period, or whether more or less significant signs of pit damage are encountered. As discussed in the previous text, there is substantial evidence that pit membrane damage occurs; however, it is not yet clear how common this phenomenon is. It is possible that damage accumulates slowly over time, but because old wood is gradually replaced by newly formed wood, vulnerability of the bulk xylem remains constant. However, under some circumstances the accumulation of damage can be so rapid that it overwhelms the capacity for xylem tissue maintenance and renewal. This brings up an interesting question: is there any coordination between the sapwood longevity, pit characteristics and intensity of external and internal stress typically encountered?

Importantly, the mechanisms potentially causing pit damage may not be mutually exclusive. On the contrary, they likely work in tandem and reinforce one another. For instance, the deforming effects of large pressure differences exerted on pit membranes may be amplified by oxidative damage caused by rising ROS concentration in xylem sap during drought. From a broader ecological perspective, pit membrane damage can provide interesting connections between abiotic and biotic stress. For instance, damage to pit membranes by pathogens may render plants more susceptible to drought. This may add another link to what already is an intricate network of feedbacks linking drought stress and pathogen outbreaks (McDowell et al., 2008; Jactel et al., 2012).

## CONCLUSIONS

There is a natural tendency to report the most “perfect looking” specimens of pits in the scientific literature. However, neglecting inherent variability in pit structure may lead to a biased view. Considering the potential importance of the rare pit hypothesis, we suggest that more attention should be given to structural irregularities, as those may represent the rare sites ultimately responsible for air-seeding.

Studying pit irregularities with SEM is challenging, and comparable to the proverbial needle in a haystack problem. Moreover, artifacts potentially associated with sample preparation represent an additional difficulty (Jansen et al., 2008). Thus, studies combining microscopic observations with hydraulic measurements (e.g., air-seeding experiments) will be necessary to move the field forward. Gaining a better understanding of the processes that lead to such irregularities provides an additional strategy for addressing this issue. We wish to emphasize that intrinsic developmental flaws and damage caused to the pit membranes by physical or chemical stress may represent two potential sources of irregularities in pit structure.

## Conflict of Interest Statement

The authors declare that the research was conducted in the absence of any commercial or financial relationships that could be construed as a potential conflict of interest.
